# The New Sydenham Society

**Published:** 1861-02

**Authors:** Charles G. Smith

**Affiliations:** 95 Washington Street, Chicago


### THE NEW SYDENHAM SOCIETY.

This is an English Society, instituted for the purpose of publishing standard works on Medical and Surgical subjects, so that they may be distributed among the largest possible number of the profession. The subscription is one guinea a year, ($5.25 U. S. currency), which entitles a member to all the publications for the current year. The works are not obtainable in our book-stores, or in any way, except by joining the Society. The following list indicates their character:

The publications for 1859:

Vol. I.—Diday "On Syphilis in Infants and Children at the Breast." Translated by Dr. Whitley.

Vol. II.—Gooch "On the most important Diseases of Women and Children," with other Papers. Woodcuts. Prefatory Essay by Dr. Ferguson.

Vol. III.—Memoirs on Diphtheria. From various French sources. Selected and translated by Dr. Semple. With a Bibliographical Appendix by Mr. Chatto.

Vol. IV comprises the two works of Professor Schroeder Van Der Kolk, (1st) "On the Spinal Cord," and (2nd) "On the Medulla Oblongata," and "On the Proximate Cause and Rational Treatment of Epilepsy." Translated by Dr. W. D. Moore, of Dublin. With numerous Lithographs.

Vol. V contains translations of the following Monographs:

1st. Kussmaul and Tenner's "Experimental Researches on the Effects of Loss of Blood in inducing Convulsions." Translated by Dr. Bronner, of Bradford.

2nd. Wagner "On the Resection of Bones and Joints." Translated by Mr. T. Holmes. Numerous Woodcuts.

3rd. Professor Graefe's Three Papers on Glaucoma, Iridectomy, &c., &c. Translated by Mr. T. Windsor, of Manchester.

The publications for 1860:

Vol. VI—Clinical Memoirs on Abdominal Tumors and Intumesence. By Dr. Bright. Collected and reprinted from the Guy's Hospital Reports. Edited by Dr. Barlow. With numerous Woodcuts.

Vol. VII.—A Year-Book for 1859. The Year-book is designed to form a Register in condensed abstract of all Important Communications, whether British or Foreign, in Medicine, Surgery, and their allied Sciences during the year. It will consist of five sections:—1st. Anatomy and Physiology. Edited by Dr. Harley. 2nd. Medicine. Edited by Dr. Handfield Jones. 3rd. Surgery. Edited by Mr. Hulke. 4th. Diseases of Women and Children. Edited by Dr. Graily Hewitt. And 5th. Forensic Medicine and Toxicòlogy. Edited by Dr. Odling.

Vol. VIII.—Frerich's Clinical Account of Diseases of the Liver. Vol. 1. With Woodcuts. Translated by Dr. Murchison.

Vol. IX.—Vogel and Neubauer on the Examination of the Urine. A Manual intended for the assistance of the practical physician. With Woodcuts and Plates.

Vol. X.—The First Fasciculus of an Atlas of Illustrations of Diseases of the Skin, copied from those of Hebra, and of Life-size.

The works for 1860, and probably those for 1859, may yet be had by subscribing for those years. The subscription for 1861 is now due and should be paid in advance, in order to secure the publications of the year. This pre-payment is essential to the efficient and prompt action of the Society in publishing the books, and relieves from embarrassing uncertainty. As it is a mutual benefit association, it is hoped that members of the profession will take interest enough in it, to mention it to their friends. The expenses of exchange and carriage are to be divided, among the local members. The circulars of the Society may be obtained, and specimens of books seen, at the office of Dr. V. L. Hurlbut, 122 Randolph street; or at the office of the undersigned, who is authorized to forward subscriptions,

CHARLES G. SMITH, M. D.,

95 *Washington Street*, *Chicago.*

